# Bacterioplankton taxa compete for iron along the early spring–summer transition in the Arctic Ocean

**DOI:** 10.1002/ece3.11546

**Published:** 2024-06-18

**Authors:** Fernando Puente‐Sánchez, Luis Alberto Macías‐Pérez, Karley L. Campbell, Marta Royo‐Llonch, Vanessa Balagué, Pablo Sánchez, Javier Tamames, Christopher John Mundy, Carlos Pedrós‐Alió

**Affiliations:** ^1^ Department of Systems Biology Centro Nacional de Biotecnología, CSIC Madrid Spain; ^2^ Microbial Ecology Division, Department of Aquatic Sciences and Assessment Swedish University of Agricultural Sciences Uppsala Sweden; ^3^ UiT The Arctic University of Norway Tromsø Norway; ^4^ Centre for Earth Observation Science, University of Manitoba Winnipeg Manitoba Canada; ^5^ Department of Marine Biology and Oceanography Institut de Ciències del Mar, CSIC Barcelona Spain; ^6^ Present address: Department of Aquatic Sciences and Assessment Swedish University for Agricultural Sciences (SLU) Uppsala Sweden; ^7^ Present address: Department of Evolutionary and Integrative Ecology Leibniz Institute of Freshwater Ecology and Inland Fisheries (IGB) Berlin Germany; ^8^ Present address: UiT The Arctic University of Norway Tromsø Norway

**Keywords:** Arctic Ocean, bacteroidetes, competition, gammaproteobacteria, iron

## Abstract

Microbial assemblages under the sea ice of the Dease Strait, Canadian Arctic, were sequenced for metagenomes of a small size fraction (0.2–3 μm). The community from early March was typical for this season, with *Alpha*‐ and Gammaproteobacteria as the dominant taxa, followed by Thaumarchaeota and Bacteroidetes. Toward summer, Bacteroidetes, and particularly the genus *Polaribacter*, became increasingly dominant, followed by the Gammaproteobacteria. Analysis of genes responsible for microbial acquisition of iron showed an abundance of ABC transporters for divalent cations and ferrous iron. The most abundant transporters, however, were the outer membrane TonB‐dependent transporters of iron‐siderophore complexes. The abundance of iron acquisition genes suggested this element was essential for the microbial assemblage. Interestingly, Gammaproteobacteria were responsible for most of the siderophore synthesis genes. On the contrary, Bacteroidetes did not synthesize siderophores but accounted for most of the transporters, suggesting a role as cheaters in the competition for siderophores as public goods. This cheating ability of the Bacteroidetes may have contributed to their dominance in the summer.

## 1 INTRODUCTION

Polar environments are characterized by prolonged periods of ice cover and darkness in winter, generally low water temperatures, and extended irradiance and ice melting in summer (Bunse & Pinhassi, 2017). The transition from winter to spring and summer involves, thus, dramatic environmental changes, and these are reflected in the composition of the microbial assemblages inhabiting the ice and the underlying water (Alonso‐Sáez et al., 2008; Sala et al., 2008). The strategies used by Arctic microorganisms to thrive in these highly variable conditions are still poorly understood, partially due to the logistic difficulties of sampling during the early part of the year. This becomes particularly relevant as climate change is having a particularly high impact in the Arctic Ocean through the reduction in sea ice (Steele et al., 2008; Stroeve & Notz, 2018; Wang et al., 2020), and this may in turn change the distribution and cycling of the nutrients that these microorganisms need to survive (Harada, 2016; Slagstad et al., 2015).

Among these, iron is one of the most relevant, as the insolubility of the most common form, Fe (III) and the fact that its sources are of terrestrial origin (Martin & Fitzwater, 1988) make it a limiting nutrient in between 15% and 40% of the global surface ocean (Johnson et al., 1997; Moore et al., 2013). Iron is not typically considered a limiting nutrient in the Arctic Ocean (Klunder, Bauch, et al., 2012), with primary productivity being instead controlled by light under normal circumstances and by nutrients other than iron during blooms (Gosselin et al., 1990; Leu et al., 2015). However, the reduction in ice cover due to climate change has been predicted to increase light availability and in turn primary productivity, increasing the demand for iron and making it potentially limiting (Rijkenberg et al., 2018), which could have important consequences for the resident bacterioplankton. We thus sought to characterize the different iron acquisition strategies utilized by these prokaryotic communities before, during, and after the Spring–Summer ice melt transition. To achieve this, we analyzed a metagenomic and metatranscriptomic time series from the Dease Strait (Canadian Arctic) and collected data from early March to late July.

Microorganisms use a variety of strategies to acquire iron from the environment, including direct diffusion through porins, transport through specific cation channels, or the release of iron‐scavenging molecules to the extracellular environment (Krewulak & Vogel, 2008; Qiu et al., 2022; Sutak et al., 2020). Among the latter, siderophores are one of the most common molecules utilized for iron acquisition in bacterioplankton (Ahmed & Holmström, 2014; Boiteau et al., 2016; Bundy et al., 2018; Debeljak et al., 2019), as they have a very high affinity for Fe(III) and are thus efficient scavengers even when its concentration is low (Krewulak & Vogel, 2008). These small molecular weight compounds are synthesized by certain microorganisms and then released into the environment (Hopkinson & Barbeau, 2012; Manck et al., 2022; Payne et al., 2016), where they can bind to Fe(III) and later be reacquired through specific transporters. Siderophores, however, are energetically costly not only due to their relatively complex biosynthetic pathways but also to the fact that they need to be exported to the extracellular environment, where they can be potentially lost before benefiting the producer (Khan et al., 2018). Some bacteria thus benefit by not synthesizing siderophores but having the transporters acquire them from the environment. These bacteria act as cheaters, exploiting “public goods” that they have not contributed to create, leading to an inter‐dependence scenario that has been further elaborated as part of the “Black Queen Hypothesis” (Morris et al., 2012). Due to these peculiarities, the interactions between siderophore producers and nonproducers have been explored with cultures of marine *Vibrio* to illustrate competition for public goods (Cordero et al., 2012) and the evolutionary implications of these interactions have been reviewed (Kramer et al., 2020). Most experimental information, however, comes from studies with pure cultures of Proteobacteria, and there is still limited information on how producer–consumer dynamics work in nature, where the large diversity of coexisting microorganisms may result in complex interactions (Zoccarato et al., 2022).

In the Arctic, Bacteroidetes becomes the most abundant taxon of the assemblage in summer (Ghiglione et al., 2012; Pedrós‐Alió et al., 2015). On the other hand, *Proteobacteria*, which are the most abundant taxa in winter and early spring, decrease in abundance (Pedrós‐Alió et al., 2015). One remarkable difference between Proteobacteria and Bacteroidetes is their capability to synthesize siderophores. Proteobacteria have been shown to synthesize dozens of different siderophores (Vraspir & Butler, 2009), while almost no instances of siderophore synthesis are known among Bacteroidetes (Guan et al., 2013). However, Bacteroidetes are known to have receptors for iron‐siderophore complexes (D'Onofrio et al., 2010; Zhu et al., 2020). Therefore, we examined the time evolution of the bacterioplankton assemblage and the presence and abundance of iron acquisition genes through the ice melting period with the aim of testing whether the iron acquisition strategies may have a role in the outcome of the competition among taxa.

## 2 MATERIALS AND METHODS

### 2.1 Sampling

Samples were collected in the Dease Strait, lower Northwest Passage, Nunavut, Canada (69.03° N, 105.33° W; Figure S1). The water depth was 60 m, and samples were collected between 7 March and 24 June 2014, as part of the 2014 Ice‐Covered Ecosystem‐CAMbridge Bay Process Study (ICE‐CAMPS). During the ICE‐CAMPS campaign, Dease Strait was covered by first‐year ice (1.8–2.1 m thick) with drifted snow ranging from 5 to <25 cm in depth, until melt commenced on 8 June. Snow melt caused the ice surface to flood by 16 June (Diaz et al., 2018), followed by subsequent drainage and the formation of melt ponds that persisted until the ice breakup on 19 July. One week after the ice breakup, the site was sampled for the final time in ice‐free waters on 30 July aboard the *R/V Martin Bergmann*.

Water samples were collected opportunistically thirteen times at a depth of 2.5 m, through a 20‐cm‐diameter hole from 7 to 18 March 2014, protected by a portable tent. For the remainder of the ice‐covered season (21 April to 24 June 2014), samples were taken from a 2‐m^2^ sampling hole maintained within a heated tent. Finally, the 30 July sample was collected, as mentioned, from a ship. A submersible water pump mounted on an under‐ice arm was used to collect the ice‐water interface at 2.5 m. Hydrographic profiles were also taken using a conductivity, temperature, and depth probe (RBR XR‐620 CTD) equipped with a chlorophyll (chl) *a* in vivo fluorescence sensor. The region is in close proximity to mainland Canada and receives freshwater inputs from the nearby Coppermine, Hood, Burnside, and Ellice rivers (Figure S1). Further details about the study site can be found in Campbell et al. (2015, 2016, 2017).

### 2.2 Physico‐chemical measurements

Approximately 10 m from the location of water sampling, profiles of under‐ice photosynthetically active radiation (PAR) were collected between 10:00 a.m. and 12:30 p.m. local time. This was done using a spherical underwater sensor (LI‐193 LI‐COR) lowered through an auger hole that took measurements at 1‐m depth intervals from the ice‐water interface down to 20 m. During measurements, the sampling hole was covered to limit contamination by surface radiation. The disturbance of these sites for light measurement was kept to a minimum, and the hole was approached along a single path from the north.

Following transport to laboratory facilities, water samples that were kept in darkness close to, but not below, −2°C, were immediately processed for chl *a* and nucleic acids. Subsamples for chl *a* were filtered onto GF/F filters (Whatmann) before subsequent pigment extraction in 10 mL of 90% acetone for 24 h in darkness and at 4°C. Fluorescence was measured before and after acidification with 5% HCl (Turner Designs Trilogy Fluorometer) according to Parsons et al. (1984), and chl *a* concentration was calculated using the equations of Holm‐Hansen et al. (1965).

In order to collect nucleic acids, approximately 10 L of water was prescreened to remove visibly identifiable plankton and then filtered through a tiered stainless steel large‐volume filtration system (Millipore) with a succession of 20, 3, and 0.22 μm filters. Separate filters were prepared for RNA and DNA (filtering 10 L for each). Filters were immediately frozen at −80°C. Here, we present data from the 0.22 μm filters only.

### 2.3 Nucleic acid extraction

DNA was extracted according to the phenol/chloroform protocol detailed by Massana et al. (2004). After thawing, frozen filters were cut into small pieces and placed into sterile cryovials. Lysis buffer and lysozyme (final concentration: 1 mg mL^−1^) were added to the cryovials, and they were incubated at 37°C for 45 min. Sodium dodecyl sulfate (10%) and Proteinase K (0.2 mg mL^−1^) were added, and the mixture was incubated at 55°C for 1 h. The lysate was mixed twice with an equal volume of phenol/chloroform/isoamyl alcohol (25:24:1, pH 8) and centrifuged at 12,000× rpm (10 min). A second extraction with chloroform/IAA (24:1) was carried out to remove the residual phenol. The aqueous phase containing the DNA was concentrated and purified by cleaning up with sterile purified water using Amicon Ultra Centrifugal filters (Millipore). The DNA yield and integrity were quantified in a Nanodrop spectrophotometer (NanoDrop 1000 Thermo Fisher) and a Qubit fluorimeter (Thermo Fisher). DNA extracts were stored at −80°C.

RNA was extracted after cutting the frozen filters into small pieces and using Qiagen's RNeasy kit. After a DNase treatment with Ambion's Turbo DNA‐free kit, RNA yield and integrity were quantified with a Nanodrop spectrophotometer (NanoDrop 1000 Thermo Fisher) and a Qubit fluorometer (Thermo Fisher). The RNA extracts were stored at −80°C. Prior to sequencing, ribosomal RNA depletion was performed by Sequentia Biotech (http://www.sequentiabiotech.com/) using Illumina's TruSeq Stranded Total RNA LT – with RiboZero.

### 2.4 High‐throughput sequencing (HTS)

DNA samples were sequenced in two batches. The first batch included most metagenomic samples and was sequenced at CNAG (https://www.cnag.crg.eu) on the Illumina HiSeq2000 sequencing platform using a TruSeq paired‐end cluster kit, v3. The second batch included four metagenomic samples (May 10, June 1, June 15, and July 30) and all metatranscriptomic samples. The number of reads per sample is shown in Table S1. We used the SqueezeMeta pipeline (v1.3) and the SQMtools R package (Puente‐Sánchez et al., 2020; Tamames & Puente‐Sánchez, 2019) for all the bioinformatics analyses. Details of the assembly are shown in Table S2. Average gene copy numbers per genome were calculated as described in Puente‐Sánchez et al. (2020) using the median coverage of 10 universal single‐copy marker genes (Salazar et al., 2019). These markers are particularly suitable for normalizing metagenomic and metatranscriptomic data to provide estimates of relative per‐cell gene copies because they represent constitutively expressed single‐copy housekeeping genes (Salazar et al., 2019). Furthermore, since average copy numbers are normalized against the coverage of well‐conserved genes, they should be unaffected by the percentage of mapped and/or annotated reads, making them better to compare the abundances of the same function in different samples. Thus, throughout the manuscript, we will use average copy numbers per genome when discussing the normalized abundance of individual functions in the microbiome, and percentages of reads assigned to each taxon when discussing taxonomic distributions. Sequences were deposited under NCBI BioProject ID PRJNA803814.

### 2.5 Taxonomic assignment of ORFs and contigs

For each ORF DIAMOND (v0.9.22), homology searches were performed with its amino acid sequence against the GenBank nr database (downloaded in September, 2019). An e‐value cutoff of 1e‐03 was set to discard poor hits. The best hit was obtained, and then we selected a range of hits (valid hits) having at least 80% of the bitscore of the best hit and with an identity to the nr database no smaller than 10% of the identity of the best hit. The Last Common Ancestor (LCA) of all these hits was obtained at diverse taxonomic ranks (from phylum to species). In order to allow for some flexibility in the case of putative transfer events or incorrect annotations in the database, we reported a taxon as the LCA as long as it was supported by 90% of the valid hits.

To ensure trustworthy annotations, we also required a minimum identity for the nr database in order to assign ORFs to the different taxonomic ranks. These identity cutoffs were based on Luo et al. (2014) and were 85%, 60%, 55%, 50%, 46%, 42%, and 40% for the species, genus, family, order, class, phylum, and superkingdom ranks, respectively.

Finally, for each contig, we obtained a consensus taxonomy from the annotations of all the ORFs encoded in the contig, such as 50% of all the genes (regardless of whether they could be annotated or not) and 70% of the annotated genes belonging to the same taxon. After mapping the reads to the contigs using Bowtie2 v2.3.4.1 (Langmead & Salzberg, 2012), contig taxonomies were used for calculating global taxon abundances in our samples, while ORF taxonomies were used to calculate the taxonomic distribution of particular functions.

### 2.6 Functional annotation of open reading frames (ORFs)

The ORFs were functionally annotated into Clusters of Orthologous Groups (COGs) by downloading the eggNOG database (Huerta‐Cepas et al., 2016), selecting the entries annotated as COGs, creating a DIAMOND database and using it for annotation as described in Tamames and Puente‐Sánchez (2019). COGs were used for functional annotation instead of KEGG orthologs (KOs) or PFAMs, since their finer granularity allowed for a more careful curation of the annotations (see next section).

### 2.7 Identification of siderophore synthesis genes and pathways

Pathways for siderophore synthesis usually involve two types of enzymes (Crosa & Walsh, 2002). In the first steps, fairly common enzymes are used, for example, the protein catalyzing the step from chorismate to isochorismate. These enzymes are coded by genes that are common not only to several siderophore synthesis pathways but also to more general central metabolic pathways. And next, several additional steps that usually require nonribosomal peptide synthetases (NRPS), polyketide synthases, and NRPS‐independent siderophore synthetases (NIS) that condense small and large molecules until the complex structures of the siderophores are completed. In many cases, several of these NRPS are common to different pathways, and, thus, different gene names are classified in the same COG category. For example, COG3486 has representatives in at least 13 siderophore synthesis pathways (see Figure S2). In more extreme cases, a single COG category may conflate NRPS involved in siderophore biosynthesis with NRPS related to the biosynthesis of other secondary metabolites such as antibiotics or toxins. Thus, detecting this COG in metagenomes does not indicate which siderophore pathway is present. On the contrary, some genes are assigned to more than one COG, further complicating the issue. For example, gene *dhbE* in the bacillibactin synthesis pathway is assigned to COGs 0318 (AMP‐dependent synthetase and ligase) and COG1021 (2,3‐dihydroxybenzoate‐AMP ligase). We manually checked all the siderophore synthesis pathways in MetaCyc (Caspi et al., 2020) and added a few more (Table S3). We have summarized this complexity in a network (Figure S2) that will facilitate the identification of both common and unique genes in the synthesis pathways of the different siderophores.

Since identifying the siderophore pathways that are present in metagenomes and expressed in metatranscriptomes is complicated, we adopted the following criteria:

1. In order to state that the pathway for a given siderophore MAY BE present, all the genes in the pathway must be present.
2. In order to state that the pathway IS present, besides presence of all the genes, at least one of the genes must be unique to this pathway.
3. If at least one gene of a pathway is absent, we consider the whole pathway absent.

There was an additional difficulty. A substantial portion of the genes involved in siderophore synthesis belonged to COG0318. This COG includes genes *entE* in the salmochelin and enterobactin pathways (three different steps in the pathways), *pvdI* and *pvdL* in pyoverdin (first condensation step), *dhbE* in bacillibactin (two successive steps), *asbC* in petrobactin (a ligase in two steps), and *acsA* in achromobactin (synthase in four steps). It also includes several enzymes unrelated to siderophore synthesis. Thus, we considered the presence of this COG necessary to fulfill criterion 1 above. However, we did not count the reads assigned to this COG in the quantitation of siderophore synthesis genes because many of them might code for genes in other metabolic routes. We followed a similar procedure for the rest of the COGs, using for quantitation only those that could be unambiguously assigned to siderophore biosynthesis pathways using the high‐quality, manually curated SwissProt database (Bateman et al., 2021). Some of them, however, should be involved in siderophore synthesis. Therefore, our counts of siderophore synthesis genes are underestimates. With these very strict criteria, we could confidently state that pathways for eight siderophores were present: alcaligin, enterobactin, mycobactin, myxochelin, petrobactin, salmochelin, and staphyloferrins A and B, and that four were absent (pyochelin, pyoverdin, yersiniabactin, and amphibactin).

## 3 RESULTS

### 3.1 Changes in ecosystem parameters

**FIGURE 1:**
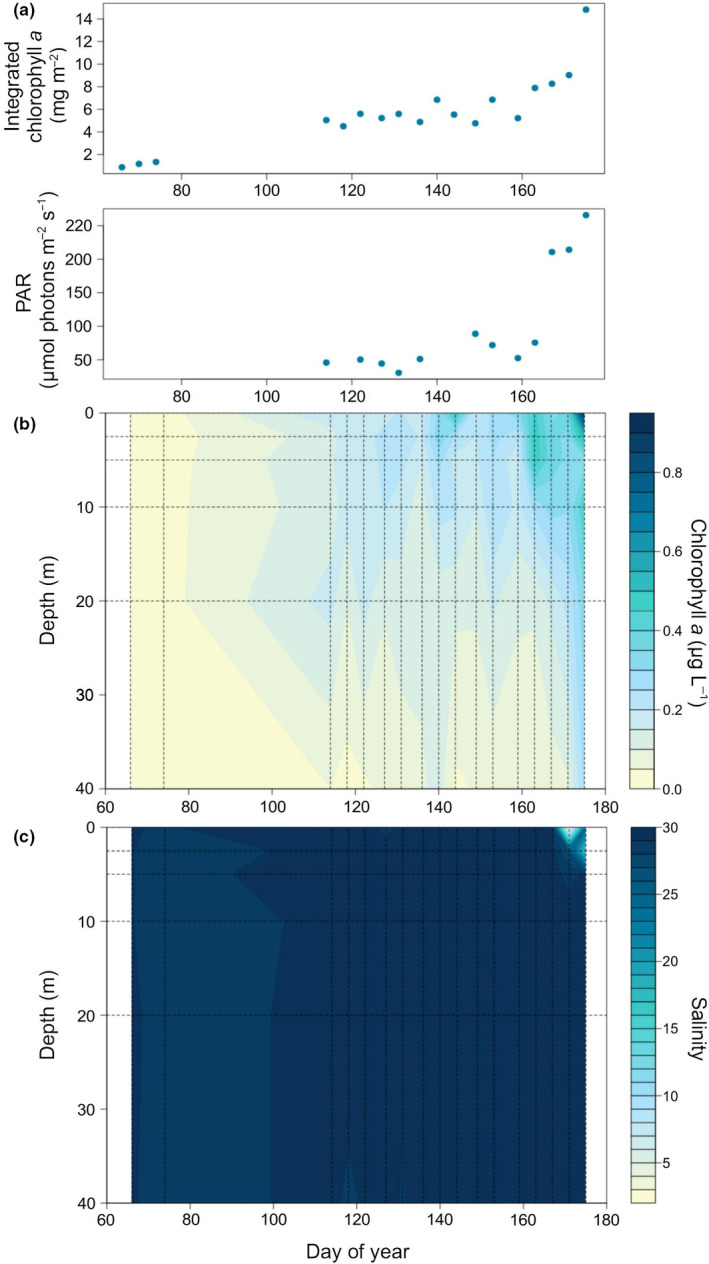
(a) Integrated chlorophyll *a* (mg m^−2^) and PAR (μmol photons m^−2^ s^−1^) along day of year. (b) Time‐depth diagram of chlorophyll *a* (μg L^−1^). (c) Time‐depth diagram of salinity.

Daylight increased from <12 h during early March to 24 h on 21 May. The consequent increase in surface irradiance was intensified by the progressive melt of surface snow and ice, with PAR transmitted to 2.5 m depth increasing from ~50 to ~220 μmol photons m^−2^ s^−1^, first steadily until 12 June, and then nonlinearly (Figure 1a; Table S6) as melt ponds formed on the surface. Chl *a* increased steadily throughout the study following the increase in PAR, while salinity decreased slightly at the surface due to ice melting (Figure 1b,c; Tables S7 and S8).

### 3.2 Taxonomic composition of the prokaryotic assemblage

**FIGURE 2:**
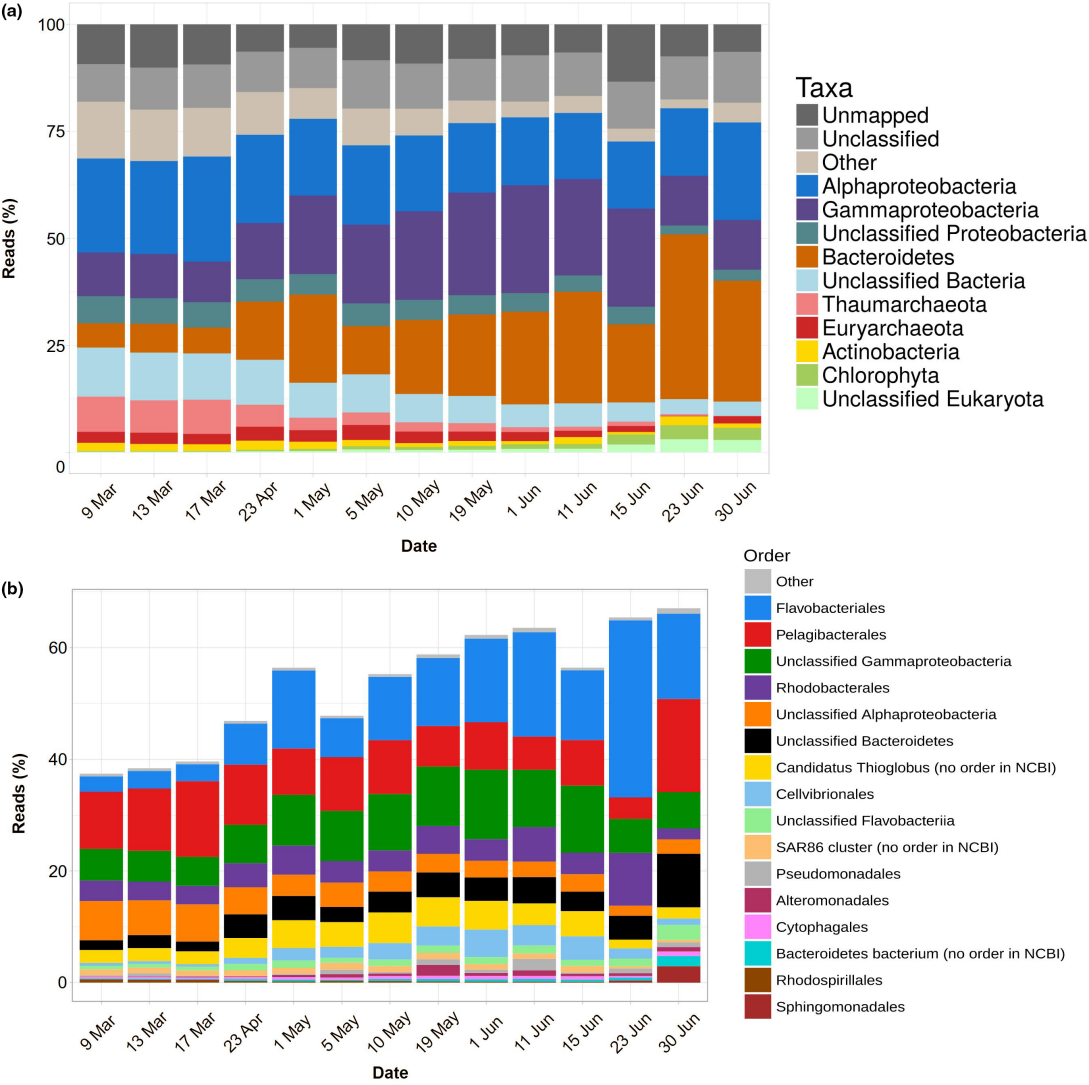
Percent of reads in metagenomes at two different taxonomic levels: phyla and Proteobacteria classes (a) and orders (b).

Microbial populations changed throughout the study period (Figure 2a). In early March, Proteobacteria dominated the assemblage. Especially, Alphaproteobacteria accounted for 36% of the reads and Gammaproteobacteria for 20%. The two other main groups were Taumarchaeota (12%) and Bacteroidetes (9%). Other minor components were Deltaproteobacteria (5%), Actinobacteria (4%), Betaproteobacteria (2.5%), Verrucomicrobia (2.5%), Euryarchaeota (2.5%), Planctomycetes (2%), and Marinimicrobia (2%). Progressive changes eventually resulted in a different composition in June. For example, on 23 June the main groups were Bacteroidetes (55%), Alphaproteobacteria (21%), Gammaproteobacteria (18%), chloroplasts (12%), and Actinobacteria (2%).

Alphaproteobacteria were, as mentioned, the most abundant group in late winter. The most important orders were Pelagibacterales and Rhodobacterales, followed by unclassified Alphaproteobacteria and small contributions from Rhodospirillales and Sphingomonadales (Figure 2b). This is a fairly usual composition in the oceans.

Gammaproteobacteria increased up to 1 June, when they reached 37% of the reads, and decreased slightly afterward (Figure 2a). The most abundant group could not be classified beyond the class level. The next taxa in abundance were *Cand*. Thioglobus and Cellvibrionales (Figure 2b). Another unusual group was the Order Pseudomonadales. In contrast, Gammaproteobacteria groups commonly abundant in the oceans, such as SAR86 and Alteromonadales had only small contributions.

Throughout the study, Flavobacteriales were the most abundant Bacteroidetes order, followed by unclassified Bacteroidetes, unclassified Flavobacteriia, and Cytophagales (Figure 2b). Most of the increase in Bacteroidetes was due to the genus *Polaribacter* (Figure S3). Indeed, at the beginning of the study, the Bacteroidetes reads were fairly well distributed among seven taxa: N55, NS9, *Marinoscillum*, NS4, *Polaribacter*, *Owenweeksia*, and *Sufflavibacter* in order of abundance, as well as several other minor components. In late June, on the other hand, 60% of the reads belonged to *Polaribacter* and the rest to several other taxa. The increase in Bacteroidetes closely followed the phytoplankton summer bloom, as indicated both by chl‐*a* (Figure 1a) and chloroplasts (Figure 2a).

Actinobacteria were present in small amounts throughout the study. Two more bacterial taxa, Verrucomicrobia and Deltaproteobacteria, were abundant at the beginning of the time series but eventually disappeared. Likewise, the two archaeal phyla, Thaumarchaeota and Euryarchaeota, were abundant in March and almost disappeared with the seasonal progression. The bloom of eukaryotic algae in June was reflected as a slight increase in the chloroplast reads toward the end of the study period (Figure 1a).

### 3.3 Main iron transport genes

We searched for almost 250 unique genes grouped in 93 COG categories related to iron acquisition and siderophore synthesis (Table S3). We pooled the genes into groups with similar functions (Table 1, see scheme in Figure S4). Thus, the main transporters of iron were classified into: (i) ferrous iron ABC transporters, (ii) ferric iron siderophore OMT transporters (TonB dependent), and (iii) ferric iron ABC transporters. These are the proteins destined to transfer ferrous iron across the cell membrane and ferric iron bound to siderophores across both membranes. Other categories were (iv) divalent cation ABC transporters (transporting iron and other cations across the cell membrane), (v) transporters of iron bound to dicitrate, (vi) transporters of heme groups, (vii) storage of iron as ferritin, and (viii) regulators of iron metabolism. Finally, we also examined siderophore synthesis genes (see next section).

**TABLE 1:** Categories of iron transport and siderophore synthesis genes used in the present paper.

Category	Function	Transporter type	Location	Examples
Fe(II)	Ferrous iron transport	ABC	Cell membrane	*efeUOB*
Fe(III)‐OM	Ferric iron +siderophore transport	TonBD	OUTER membrane	*fhuA*
Fe(III)‐CM	Ferric iron +siderophore transport	ABC	Cell	*fhuBCD*
Divalent cation	Divalent cations transport including iron	ABC	Cell	*sitABC*
Dicitrate	Iron + dicitrate transport	TonBD, ABC	Out, cell	*fecABCD*
Heme	Iron + heme transport	TonBD, ABC	Out, cell	*hemR*, *hemUV*
Storage	Ferritin	–	Cytoplasm	*bfr*
Regulators	Regulation of iron related genes	–	Cytoplasm	*fur*, *irr*
Siderophore synthesis	Synthesis of siderophores	–	Cytoplasm	*entABCDF*

**FIGURE 3:**
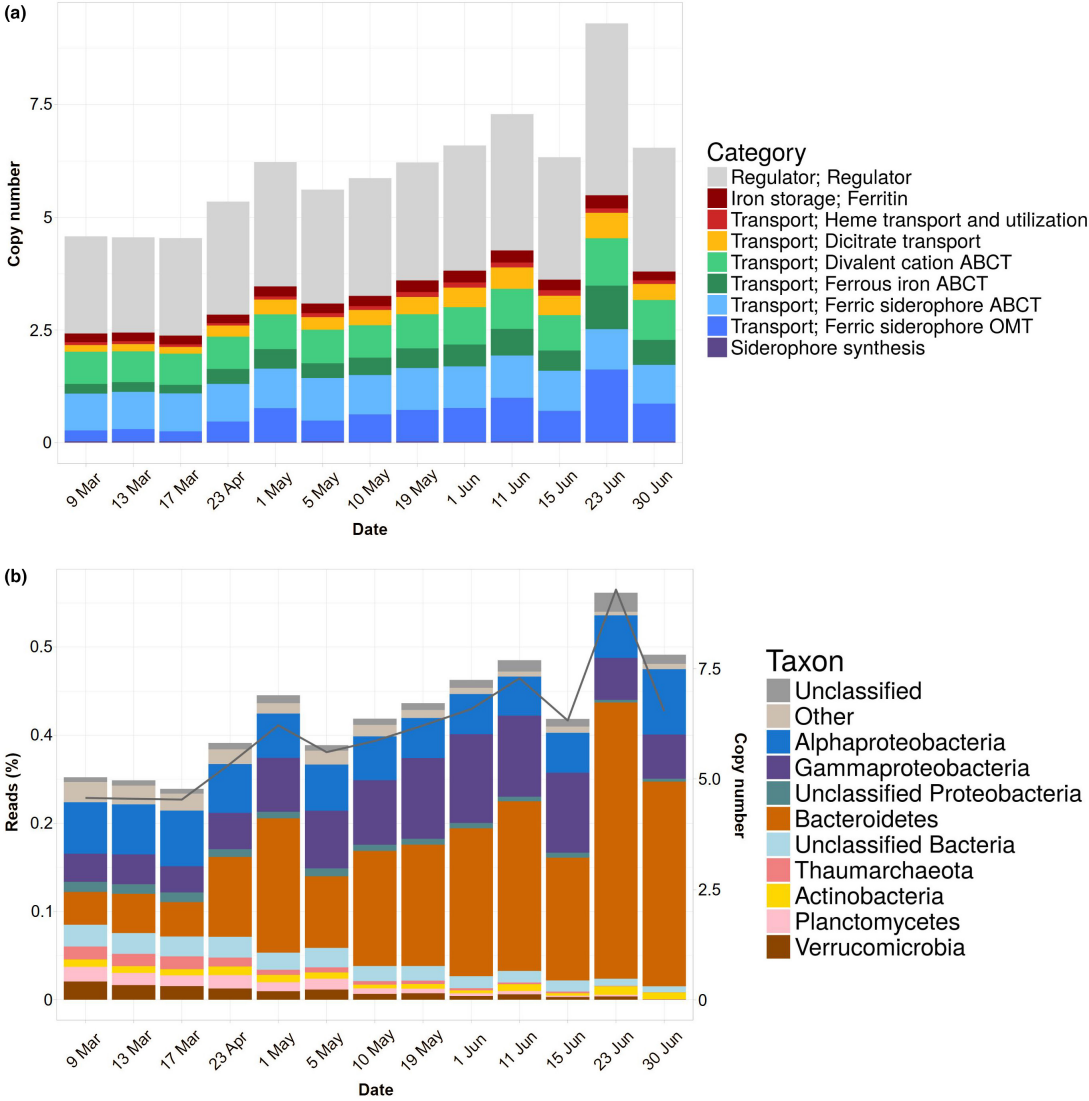
(a) Copy numbers of iron‐related genes through the study period in metagenomes. (b) Percent of the reads assigned to different taxonomic groups (bars, left hand scale) and copy numbers (line and right‐hand scale).

The normalized abundance of iron‐related genes increased from a copy number (CN) of less than 5 in March to higher than 8 in late June (Figure 3a). Thus, the CN of genes devoted to iron acquisition almost doubled along the time series. Three of our categories, ferric iron siderophore ABC transporters, storage, and siderophore synthesis, did not show appreciable changes. Two other, heme and divalent cation ABC transporters increased moderately, while ferric siderophore OMT transporters, dicitrate, and ferrous iron ABC transporters increased substantially. Iron uptake regulation genes also increased and were those with the highest CN throughout the study (between 2 and 4; Figure 3a). Globally, regulator genes accounted for about half of the copies in the category, ferric siderophore transporters (OMT and ABC) for a quarter and the remaining genes for another quarter.

The taxonomy of these genes generally corresponded to that of the community (compare Figures 2a and 3b). In other words, the most abundant taxa were responsible for most of the iron‐related genes. There was only one obvious difference: while Alphaproteobacteria were always the first or second group in abundance, they were always the third one when considering only the iron‐related genes, with Gammaproteobacteria and Bacteroidetes alternating as those with the most genes. This underrepresentation was likely due to the fact that the most abundant member of the Alphaproteobacteria was the order Pelagibacterales (Figure 2b), which is known to lack all TonB‐dependent transporters and to have a very small percent of their genome devoted to iron acquisition genes (Marta Cobo‐Simón, Javier Tamames and Carlos Pedrós‐Alió, Unpublished data).

### 3.4 Ferric iron transport genes

**FIGURE 4:**
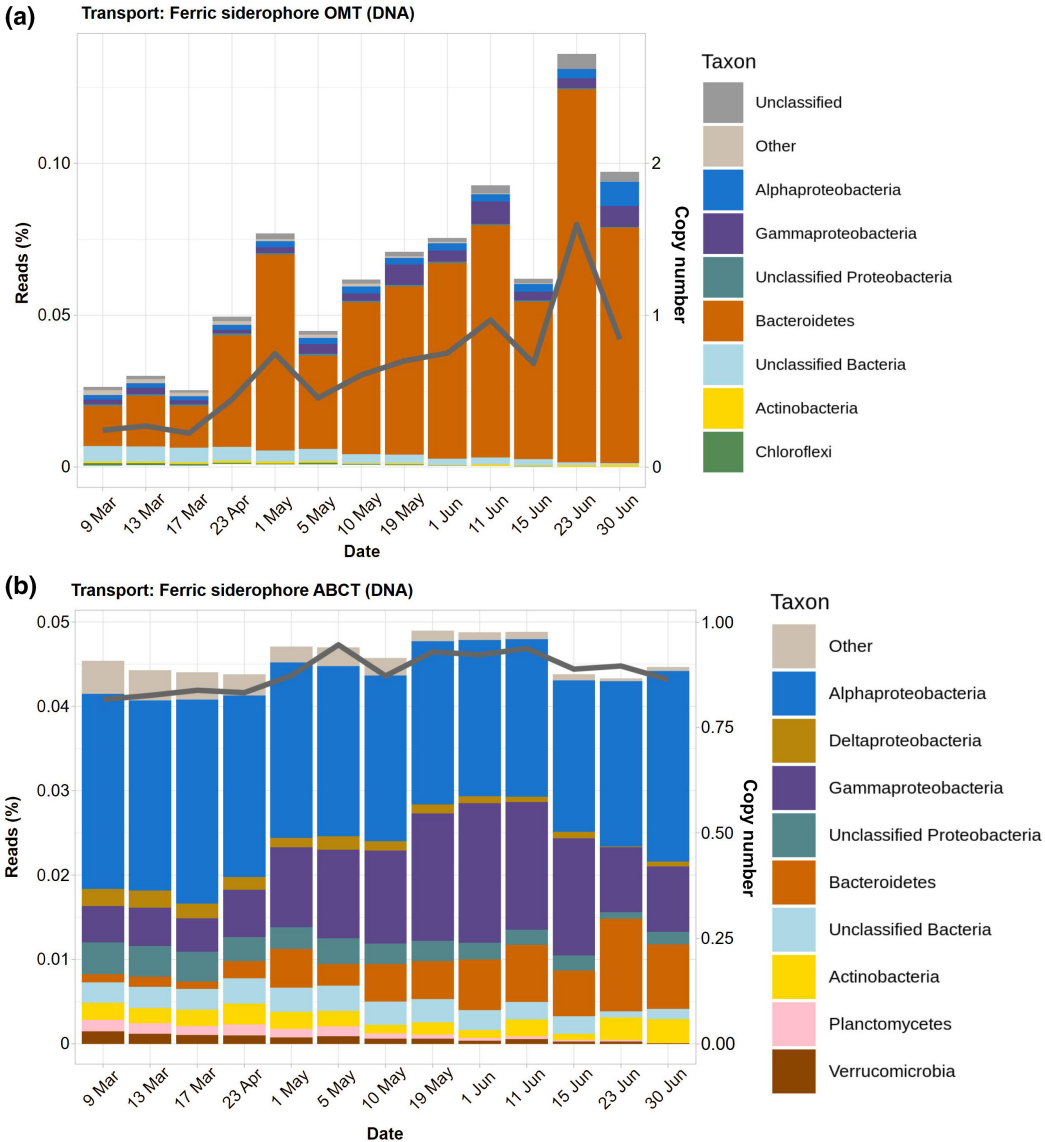
Taxonomic assignments and copy numbers of ferric iron transporter genes along the study: outer membrane ferric siderophore TonBD (above) and cell membrane ABC (below).

There were large differences in abundance and taxonomy between the outer membrane and cell membrane transporters (Figure 4a,b). The TonB‐dependent ferric siderophore OMT transporters increased from an average CN of 0.3 in March to reach a maximum of 1.6 in late June. As some of the COGs related to the TonB‐dependent ferric siderophore OMT transporters can also include transporters for other substances, such as cobalamin, we also focused on COG1629 in particular, which, to our knowledge, is in the large majority of cases diagnostic for siderophore transport. COG1629 showed a similar trend in our data, increasing from a CN of 1.48 in March to a maximum of 5.20 in late June (Table S5).

Most of this increase was due to Bacteroidetes (Figure 4a). In effect, this group accounted for 90% of the reads of these genes in late June. On the contrary, the inner membrane ferric siderophore ABC transporters remained almost constant around a CN of 0.9. The taxonomy was also completely different. In order of importance, the main groups were Alpha‐, Gammaproteobacteria, and Bacteroidetes in third place (Figure 4b).

An alternative path to obtaining ferric iron is to incorporate it into dicitrate. The genes for this process increased in parallel to the TonB‐dependent ferric siderophore OMT transporters, although at lower CNs (always below 0.6, Figure S5a). In this case, Gammaproteobacteria and Bacteroidetes were responsible for most of the reads.

The last ferric iron transporter genes that we found were those devoted to transporting heme‐bound iron (Figure S5b). These genes also increased with time, mostly due to the Gammaproteobacteria. Bacteroidetes did not have these genes. And toward the end of the study, Actinobacteria and Chlorophyta showed an increased contribution. The CNs, however, were low, always below 0.15.

### 3.5 Siderophore synthesis genes

**FIGURE 5:**
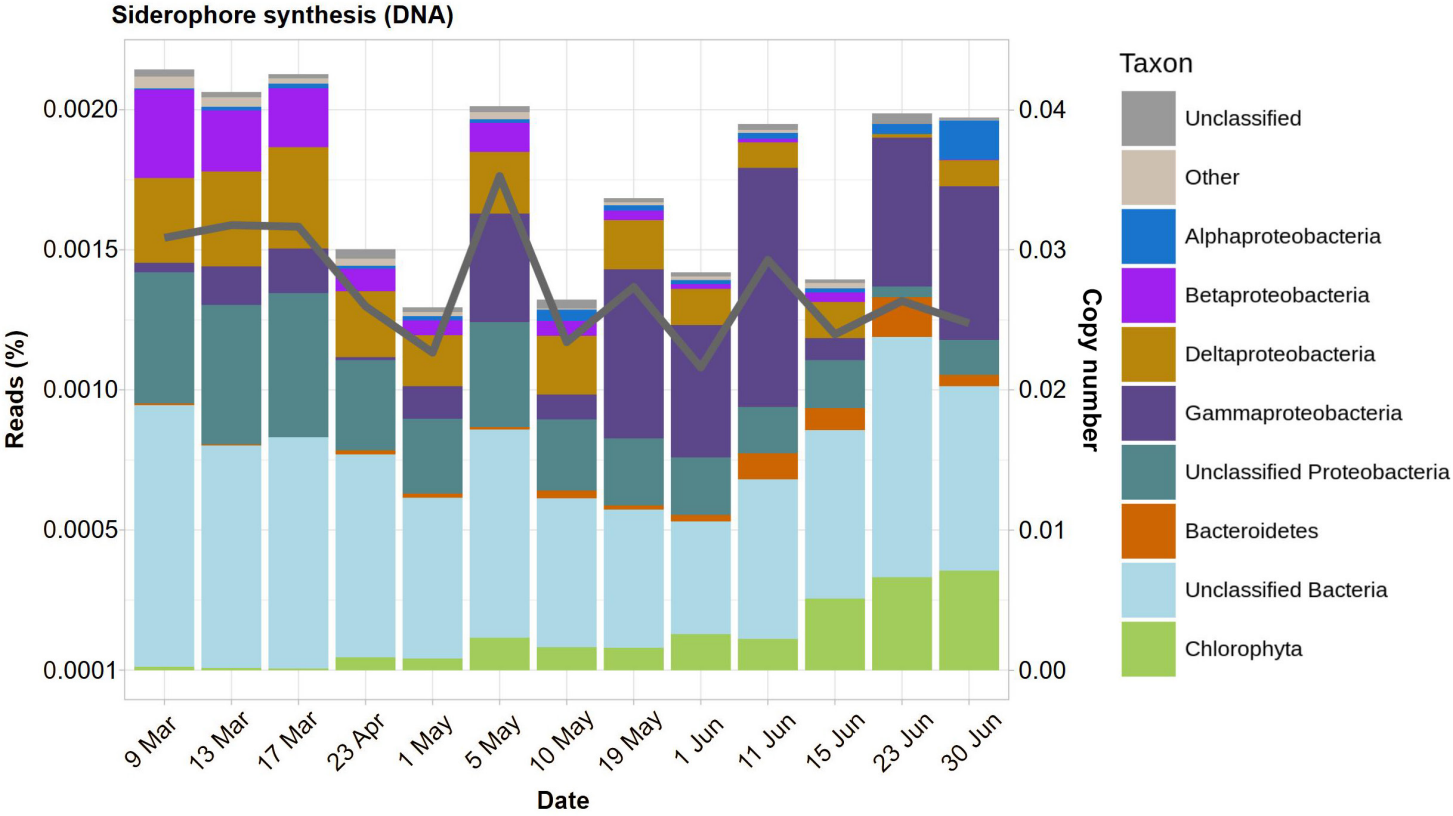
Taxonomic assignments (bars) and copy numbers (line and right scale) of siderophore synthesis genes.

The CN of siderophore synthesis genes oscillated slightly around 0.025 (Figure 5). These values are likely underestimated because we excluded all the reads of COG0318 (see M&M). Still, the CNs are very low compared to the genes that incorporate iron‐bound siderophores. Thus, the fraction of the community capable of siderophore biosynthesis seemed to be moderate and more or less constant. A portion of these genes could not be assigned to particular bacterial groups. But of those that could be assigned, most belonged to Proteobacteria. Interestingly, Beta‐ and Deltaproteobacteria were significant contributors in March, while Gamma‐ and, to a lesser extent, Alphaproteobacteria were the main groups in June. A striking difference with previous groups of genes was that Bacteroidetes almost did not contribute anything to siderophore synthesis.

With the criteria explained in M&M, we could confidently state that pathways for eight siderophores were present: alcaligin, enterobactin, mycobactin, myxochelin, petrobactin, salmochelin, and staphyloferrins A and B, and that four were absent (pyochelin, pyoverdin, yersiniabactin, and amphibactin). Again, considering our criteria, the presence of the remaining studied siderophores could not be confirmed (Table S4). All eight siderophore synthesis pathways identified as present were found in all the metagenomes except the earliest one on 9 March (Table S5).

### 3.6 Ferrous iron transport genes

**FIGURE 6:**
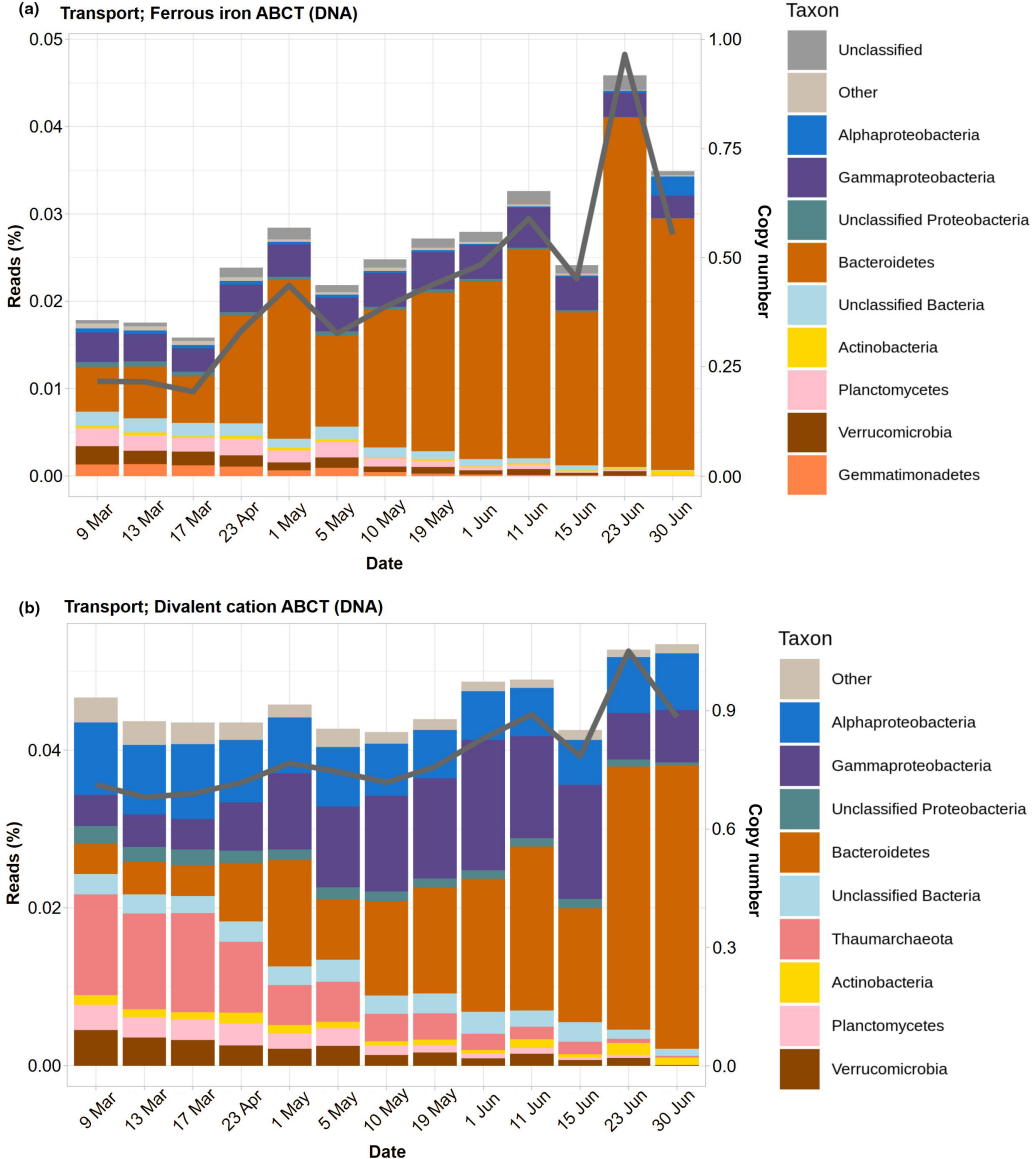
Taxonomic assignments and copy numbers of (a) ferrous iron ABC transporter genes and (b) divalent cation ABC transporter genes along the study.

Ferrous iron is able to diffuse through porins into the periplasmic space. There it can be transported to the cytoplasm by ABC transporters, either iron‐specific or able to transfer several divalent cations, including ferrous iron. The ferrous iron‐specific ABC transporters increased during the study from a copy number of 0.25 to almost 1 in June (Figure 6a). Again, most of the increase was due to Bacteroidetes, with Gammaproteobacteria having a constant contribution. Several other bacterial groups had significant contributions in March and disappeared along the study. These included Actinobacteria, Planctomycetes, Verrucomicrobia, and Gemmatimonadetes.

The divalent cation ABC transporters, on the other hand, increased very slightly from a CN of 0.8–1.0 (Figure 6b). Interestingly, Thaumarchaeota, Planctomycetes, and Verrucomicrobia accounted for half of the reads in March and decreased through spring. Alphaproteobacteria accounted for a constant proportion. Gammaproteobacteria increased until 15 June and then decreased again. Finally, Bacteroidetes had the largest increase, becoming dominant in June (70% of the reads).

### 3.7 Other iron‐related genes

We also looked for iron storage genes, mostly related to ferritin (Figure S6a). There was only a moderate increase in CN, from 0.2 to 0.3. Thaumarchaeota were abundant in March, while Gammaproteobacteria and Bacteroidetes were abundant in June. Finally, genes involved in the regulation of iron metabolisms were always the most abundant ones (Figure S6b), with CNs increasing slightly from 2.1 to 3.8. The three most abundant groups of bacteria were also responsible for most of the reads belonging to these genes.

### 3.8 Gene expression

Taxonomic assignments of the metatranscriptomic reads are shown in Figures S7 and S8. On several dates, more than half of the reads were either unmapped or unclassified. As a result, the information they provide is limited. Thus, we have only used the metatranscriptomes to show that, considering the mapped and classified reads, expression was proportional to the abundance of the taxa. Therefore, there were no differences among the different taxa in the level of expression.

## 4 DISCUSSION

### 4.1 Seasonal patterns in microbial community composition

Our results revealed a distinct shift in microbial community composition between winter and summer, in line with previous studies conducted in the Amundsen Gulf, situated approximately 600 km from our research site (Alonso‐Sáez et al., 2008). During winter, the community is generally dominated by Alpha‐ and Gammaproteobacteria, with Archaea playing a significant role that diminishes toward spring and summer (Alonso‐Sáez et al., 2012). Other minor but noteworthy groups in winter include Betaproteobacteria, Verrucomicrobia, and Actinobacteria, which typically decrease and even disappear by summer (Pedrós‐Alió et al., 2015). In contrast, Bacteroidetes abundance increases during spring and becomes one of the dominant taxa in summer, in parallel with the rise in photosynthetically active radiation (PAR) and chlorophyll‐a levels. The prevalence of the genus *Polaribacter* is commonly observed in polar waters (Ghiglione et al., 2012), a pattern consistent with our findings. Bacteroidetes are known to grow better when accompanying algal blooms and have an extensive array of outer cell membrane proteins to take up polysaccharides released into the water column by these algae (Fernández‐Gómez et al., 2013; Niemi et al., 2014; Pedrós‐Alió et al., 2015; Redondo et al., 2023; Reintjes et al., 2020). The prevalence of Alpha‐ and Gammaproteobacteria and Bacteroidetes, as observed in this study, resembles the typical microbial community structure found in the Arctic Ocean (Pedrós‐Alió et al., 2015). This pattern contrasts, however, with the usual community composition in temperate waters, where Alphaproteobacteria generally represent 50% of the community, followed by Gammaproteobacteria and Cyanobacteria. On the other hand, Bacteroidetes typically represent only 4% of the community and Betaproteobacteria are barely detectable in these habitats (Pedrós‐Alió et al., 2015).

### 4.2 Iron in the Arctic Ocean

Several studies have determined iron concentrations in the Arctic Ocean. Most have focused either on the Central Arctic near the Eurasian coast (Klunder, Bauch, et al., 2012; Klunder, Laan, et al., 2012; concentrations ranging between 0.4 and 0.6 nM) or in the Chuckhi Sea and the Canadian Basin (Aguilar‐Islas et al., 2013; Hioki et al., 2014; Kondo et al., 2016; Nakayama et al., 2011). In the Bering Sea, Aguilar‐Islas et al. (2008) found that iron was limiting in the outer shelf areas not influenced by ice, but it was sufficient in the outer shelf influenced by ice due to the release of dissolved iron from melting ice. To our knowledge, the study by Colombo et al. (2020) represents the only investigation conducted in the Canadian Archipelago. Their findings revealed higher iron concentrations in surface water compared to deep‐water regions to the east and west, with concentrations ranging from 0.40 to 1.91 nM above 40 m and from 1.10 to 1.15 between 70 and 100 m. The authors also noted the well‐known inverse correlation between iron concentration and salinity, attributed to the influence of freshwater river discharge and ice melt enriching seawater with iron. Therefore, iron levels in this area exceed those found in most of the ocean. Moreover, N:P ratios in the ice‐water interface at our study site suggested nitrogen limitation (Campbell et al., 2016). Consequently, iron is not expected to be a limiting factor in these waters, although climate change may make it limiting in the future (Rijkenberg et al., 2018).

### 4.3 Seasonal patterns in iron uptake

While iron in ocean water could not be directly sampled in this study, our metagenomic analysis revealed the presence of iron acquisition genes across all three dominant bacterial taxa, with average copy numbers per genome greater than 1 in certain cases. Additionally, our results indicate an increased abundance of iron uptake genes (ferric siderophore OMT, ferrous iron ABCT, and regulators) during late spring, when iron concentration is expected to be higher due to riverine input and icemelt (Klunder, Bauch, et al., 2012; Klunder, Laan, et al., 2012).

Numerous studies have investigated microbial iron uptake in the waters surrounding the subantarctic Kerguelen Islands, where the windward side is iron‐depleted and the leeward side is naturally enriched in iron. For instance, Obernosterer et al. (2015) observed that heterotrophic bacteria were limited by iron in both regions during spring, even at the iron‐enriched site. Debeljak et al. (2019) analyzed the expression of several iron acquisition genes in the same area and found that transcripts for Fe(II) and Fe(III) uptake were more prevalent at the iron‐depleted stations, whereas the highest expression of siderophore‐uptake genes was observed in one of the iron‐enriched study areas (Debeljak et al., 2019). Seasonal variations in dissolved iron concentration and iron uptake gene expression have also been reported in this region. For example, Debeljak et al. (2023) noted higher dissolved iron levels during early spring compared to the late summer, along with a corresponding increase in the relative abundance of iron uptake proteins, including Fe(II), Fe(III), and siderophore OMT transporters. Similarly, Park et al. (2023) reported a significant increase in dissolved iron concentration and siderophore‐uptake transcripts from April to June in the North Pacific Ocean.

Sporadic iron inputs can result in rapid biological utilization in surface waters, notably through the production, release, and uptake of siderophores (Adly et al., 2015). Ferric siderophore OMT transporters showed indeed the greatest increase among iron uptake genes during spring in our study. Therefore, the transient supply of iron from ice melt and riverine discharge during spring may prompt bacterioplankton in the Arctic Ocean to opportunistically acquire iron through siderophores, anticipating potential periods of low iron availability.

Our results in the Arctic Ocean align with those from the iron‐enriched regions near the Kerguelen Islands, showing a prevalence of iron uptake genes with those for siderophores outnumbering those for Fe(II) and Fe(III). Therefore, it seems a general feature of polar marine bacterial assemblages that iron acquisition is a major need of bacterioplankton even under nonlimiting conditions.

### 4.4 Siderophore distribution in the coastal Arctic

In the aerobic ocean, iron predominantly exists in the Fe(III) oxidation state, characterized by its very low solubility and thus limited bioavailability. To address this challenge, marine microorganisms have evolved a number of mechanisms to acquire iron from the environment, including the uptake of Fe(III) bound to organic molecules such as siderophores (Ahmed & Holmström, 2014). Siderophores are secondary metabolites secreted by microbes that scavenge iron from the environment by forming soluble Fe(III) complexes, which are subsequently actively taken up through specific receptors (Sandy & Butler, 2009). The widespread utilization of siderophore‐mediated iron acquisition in the ocean has been substantiated by measurements of concentrations of iron‐siderophore complexes, typically constituting 0.2–10% of the total dissolved iron pool (Boiteau et al., 2016, 2019; Bundy et al., 2018; Park et al., 2023). The chemistry of siderophores in marine microorganisms has been reviewed by Sandy and Butler (2009), and Chen et al. (2019). These studies commonly employ pure culture isolates of marine bacteria, ensuring the production of siderophores in quantities suitable for detailed chemical analysis. Based on the moieties involved in iron chelation, three major types of siderophores can be distinguished: catecholates, hydroxamates, and carboxylates, with mixtures of these types being common (Chen et al., 2019). The majority of siderophore‐producing bacterial strains belong to various genera within Gammaproteobacteria, including *Halomonas*, *Vibrio*, *Marinobacter*, *Ochrobacter*, *Pseudoalteromonas*, and *Shewanella*, as well as cyanobacteria such as *Synechoccocus* (Vraspir & Butler, 2009).

Concentrations of siderophores have been systematically measured across various oceanic regions, including a vertical profile at ALOHA station off Hawaii (Bundy et al., 2018), transects from the coast offshore in the southeast Pacific (Boiteau et al., 2016), the California Current System (Boiteau et al., 2019), the North Pacific (Park et al., 2023), and along a latitudinal transect in the oligotrophic Atlantic Ocean (Mawji et al., 2008). In all these studies, hydroxamates were the predominant type of siderophores, with ferrioxamines consistently detected in surface waters and amphibactins typically more abundant in deeper waters. Extrapolating this distribution to the coastal Arctic, we would expect to find genes for ferrioxamines but not amphibactins in our 60‐m deep study region. We indeed identified the complete set of genes for ferrioxamines B and E. The biosynthesis pathway for these siderophores was thus potentially present in our samples, although, since none of these genes were unique to these pathways, we cannot assure their presence with full certainty.

The synthesis pathways for amphibactins have been well characterized in hydrocarbon‐degrading *Alcanivorax* sp. (Kem et al., 2014), while their structure, production, and utilization have also been reported in marine bacteria within the Vibrio genus (Galvis et al., 2020; Martinez et al., 2003). In our metagenomes, we found all the genes for this pathway except one. Moreover, all the genes found were common to other siderophore synthesis pathways. Therefore, according to our strict criteria, the biosynthesis pathway for amphibactin was not present in our samples. This would agree with the absence of amphibactins in surface ocean waters.

Following our conservative criteria, we identified the complete synthesis pathways for eight siderophores: four catecholates (salmochelin, enterobactin, petrobactin, and myxochelin), two hydroxamates (alcaligin and mycobactin), and two carboxylates (staphyloferrin A and B). Enterobactin and salmochelin transporters were found to be expressed in the North Pacific, indicating their utilization by marine bacteria (Park et al., 2023). However, these siderophores were not detected in seawater, and transcript abundances were low, suggesting a limited use (Park et al., 2023). Petrobactin is a common siderophore susceptible to photodegradation upon exposure to light, resulting in a reduction of Fe(III) to Fe(II) and a loss of complexation (Barbeau et al., 2002). This process likely contributes to its limited presence in surface water (Manck et al., 2022; Park et al., 2023). Moreover, Manck et al. (2022) characterized the synthesis pathway of petrobactin and demonstrated that petrobactin produced by a marine strain of Alteromonas was effective in solubilizing particulate iron for biological uptake. Hydroxamates are known to be predominant in iron‐rich seawaters, such as the North Atlantic and the California Coastal upwelling system (Boiteau et al., 2019; Mawji et al., 2008). This dominance is attributed to their high affinity for Fe(III), enabling bacteria to effectively compete for iron within mineral particles (Boiteau et al., 2019; Mawji et al., 2008).

### 4.5 Taxonomic distribution of siderophore‐related genes

Overall, the abundance of genes involved in iron uptake was proportional to the relative abundance of certain taxonomic groups. Gammaproteobacteria and Alphaproteobacteria were identified as the primary contributors to siderophore synthesis genes. In contrast, ferric siderophore transport gene abundances (OMT and ABC) were predominantly derived from Bacteroidetes and, to a lesser extent, from Proteobacteria. These results align with prior studies. For instance, Debeljak et al. (2019) found that Gammaproteobacteria and Bacteroidetes were the main taxa involved in the uptake of iron‐siderophore complexes in the Kerguelen Islands, while Alphaproteobacteria played a more significant role in Fe(II) and Fe(III) uptake. Similarly, Fourquez et al. (2016) discovered through microautoradiography that Gammaproteobacteria and Bacteroidetes were the primary bacterial taxa active in iron uptake in the same area. Moreover, in the North Pacific Ocean, Gammaproteobacteria and Alphaproteobacteria were identified as the main sources of siderophore synthesis genes, while Bacteroidetes, Gammaproteobacteria, and Alphaproteobacteria contributed significantly to siderophore transport gene abundances (Park et al., 2023).

Proteobacteria is the most extensively studied bacterial phylum concerning siderophore synthesis and uptake, particularly within pathogenic Gammaproteobacteria (Holden & Bachman, 2015; Porcheron et al., 2013). Chen et al. (2019) conducted a review of marine bacterial siderophores, identifying over 80 known siderophore families. The synthesis of these siderophores was predominantly attributed to Gammaproteobacteria, with Alphaproteobacteria, Betaproteobacteria, and Actinomycetes contributing to a lesser extent. Only one siderophore was found to be synthesized by a bacteroidetes: bisucaberin B, produced by *Tenacibaculum mesophilum* isolated from a tropical sponge (Fujita et al., 2013). Experimental evidence of siderophore synthesis in Bacteroidetes is indeed scarce. Delmont et al. (2015) identified siderophore synthesis genes within three Bacteroidetes metagenomic assembled genomes (MAGs), including two *Polaribacter* and one Cryomorphaceae, during an Antarctic bloom. Interestingly, the Proteobacteria MAGs examined in the same study lacked these genes. Moreover, some fish pathogens within the genus *Flavobacterium* have shown siderophore synthesis capabilities (Guan et al., 2013; Møller et al., 2005).

The limited contribution of Bacteroidetes to siderophore synthesis, coupled with the observed abundance of ferric siderophore OMT and ABC transporter genes, suggests that members of this taxonomic group rely on siderophores produced by other taxa, as supported by experimental data (D'Onofrio et al., 2010; Zhu et al., 2020). For instance, D'Onofrio et al. (2010) found that *Maribacter polysiphoniae*, a member of the Flavobacteriaceae family, could grow on solid agar when supplemented with a siderophore from a producer bacterium like *Microccocus luteus*, isolated from the same sediment environment. Similarly, Zhu et al. (2020) demonstrated that *Bacteroides thetaiotaomicron* could utilize enterobactin and salmochellin from *E. coli* and *Pseudomonas* in the intestinal milieu, facilitated by xusABC genes.

### 4.6 Iron‐siderophore complexes as public goods

In our Arctic study, the time series followed the development of the seasonal phytoplankton bloom (Bunse & Pinhassi, 2017), which is triggered by the increasing intensity and duration of solar radiation (Back et al., 2021). Concurrent with the seasonal phytoplankton bloom, we observed an increase in the proportion of ferric siderophore OMT transporter genes and transcriptional regulators, suggesting that iron was a desirable commodity for bacterioplankton.

Given that secreted siderophores can be shared among microorganisms from the same or different species, siderophore production is commonly viewed as a form of cooperation, wherein siderophores serve as public goods (Cordero et al., 2012; Kramer et al., 2020; Leventhal et al., 2019). While siderophore loss due to diffusion seems to deprive producers of direct benefits, it has been suggested that siderophore sharing between cells could counteract the effect of diffusion losses and be beneficial in environments where local cell densities are high (Cordero et al., 2012; Kramer et al., 2020), such as surface water during phytoplankton blooms (Ducklow et al., 2001; Jamieson et al., 2012; West et al., 2008). In this context, siderophore production might serve as an effective strategy for solubilizing iron from clumped iron particles that are introduced to the water surface during ice melt, thus enhancing nutrient accessibility for all cells in the environment (Kessler et al., 2020; Park et al., 2023; Zhang et al., 2023). In the Arctic Ocean, this process could be particularly relevant for extracting iron from humic substances discharged by rivers, given their primary contribution to the total dissolved iron pool in this region (Hioki et al., 2014; Laglera et al., 2019; Muller, 2018).

Public goods are susceptible to exploitation by cheaters, who avoid the costs of cooperation while still benefiting from the collaborative efforts of others (Butaitė et al., 2017). With respect to siderophores, there is evidence of nonproducing bacteria capable of acquiring foreign siderophores in diverse environments such as marine (Cordero et al., 2012), freshwaters (Butaitė et al., 2017), soil (Bruce et al., 2017), and host‐associated (Harrison et al., 2017), suggesting a significant role of siderophore‐mediated cooperation and cheating across bacterial communities. Notably, even microorganisms without specific transporters may still benefit from siderophores in certain contexts, such as blooms, as the photolysis of some iron‐siderophore complexes has been shown to release dissolved iron that can be acquired by the surrounding cells (Amin et al., 2009).

Our findings suggest that, while Bacteroidetes may play a minor role in siderophore synthesis, they exhibit a high abundance of ferric siderophore OMT and ABC transporter genes. This would be consistent with the aforementioned cheating scenario, wherein Bacteroidetes might exploit siderophores primarily synthesized by Gammaproteobacteria. Moreover, the low presence of siderophore synthesis genes observed in our study supports the principles of the Black Queen Hypothesis, which predicts a favored and selective reduction of genes devoted to the synthesis of public goods as long as the remaining producers are still sufficient to sustain the community (Morris et al., 2012).

### 4.7 Conclusions

Our results highlighted the potential for iron competition within the Arctic Ocean, even in an area that is commonly believed to not be iron limited. This competition seemed to increase throughout spring and early summer, in parallel with the rise of primary productivity, with the use of siderophores being an important iron acquisition strategy for the resident bacterioplankton. Our results suggest that siderophore producers (mainly gamma and alphaproteobacteria) were vastly outnumbered by consumers, with bacteroidetes becoming the most prevalent consumers in summer even though they did not contribute to production. These observations are consistent with the notion that, in the Arctic Ocean, siderophores may be a common good subjected to Black Queen dynamics. This is particularly relevant since iron limitation in the Arctic Ocean is expected to increase in the future, which may alter these dynamics and the overall biogeochemical cycling of iron in this region. Further studies, including the direct measurement of the concentrations and types of siderophores, as well as the partitioning between inorganic and siderophore‐bound dissolved iron, would be needed to fully understand the impact and future evolution of iron competition in the Arctic Ocean.

## AUTHOR CONTRIBUTIONS

**Fernando Puente‐Sánchez:** Data curation (equal); formal analysis (equal); methodology (equal); software (equal); supervision (equal); validation (equal); visualization (equal); writing – original draft (equal); writing – review and editing (equal). **Luis Alberto Macías‐Pérez:** Data curation (equal); formal analysis (equal); methodology (equal); validation (equal); visualization (equal); writing – original draft (equal); writing – review and editing (equal). **Karley L. Campbell:** Data curation (equal); methodology (equal); writing – review and editing (equal). **Marta Royo‐Llonch:** Data curation (equal); methodology (equal); writing – review and editing (equal). **Vanessa Balagué:** Data curation (equal); methodology (equal); writing – review and editing (equal). **Pablo Sánchez:** Data curation (equal); methodology (equal); writing – review and editing (equal). **Javier Tamames:** Data curation (equal); funding acquisition (equal); methodology (equal); software (equal); supervision (equal); writing – review and editing (equal). **C. J. Mundy:** Conceptualization (equal); funding acquisition (equal); writing – review and editing (equal). **Carlos Pedrós‐Alió:** Conceptualization (equal); funding acquisition (equal); supervision (equal); writing – original draft (equal); writing – review and editing (equal).

## CONFLICT OF INTEREST STATEMENT

The authors declare no conflict of interest.

## BENEFIT SHARING STATEMENT

Research collaboration was developed with scientists from Canada providing genetic samples. All collaborators are included as coauthors. The research addresses a priority concern, in this case the dynamics of marine bacteria throughout warming in the Arctic Ocean. More broadly, our group is committed to international scientific partnerships, as well as institutional capacity building.

## Supporting information


Figure S1.

Table S1.

## Data Availability

The metagenomics and metatranscriptomics datasets generated and analyzed during the current study are available on the SRA repository (BioProject ID PRJNA803814). Ecological metadata are available in the [Link], [Link] of this article.
